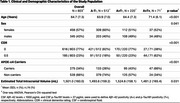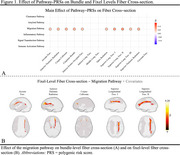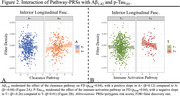# Polygenic pathways shape white matter vulnerability to Alzheimer's disease pathology

**DOI:** 10.1002/alz70856_098970

**Published:** 2025-12-24

**Authors:** Mario Tranfa, Leonard Pieperhoff, Giuseppe Pontillo, Emma S. Luckett, Lyduine E. Collij, Tiago Gil Oliveira, Niccoló Tesi, Natalia Vilor‐Tejedor, Andre Altmann, Luca Roccatagliata, Matteo Pardini, Pierre Payoux, Pablo Martínez‐Lage, Craig Ritchie, Juan Domingo Gispert, Arturo Brunetti, Henk Mutsaerts, Alle Meije Wink, Frederik Barkhof, Luigi Lorenzini

**Affiliations:** ^1^ Amsterdam University Medical Center (Amsterdam UMC), Amsterdam, North Holland, Netherlands; ^2^ University College London (UCL), London, United Kingdom; ^3^ Department of Advanced Biomedical Sciences, University “Federico II”, Naples, Italy; ^4^ Amsterdam UMC location VUmc, Amsterdam, Netherlands; ^5^ Clinical Memory Research Unit, Department of Clinical Sciences Malmö, Faculty of Medicine, Lund University, Lund, Sweden; ^6^ School of Medicine, Institute of Life and Health Sciences (ICVS), University of Minho, Braga, Portugal; ^7^ Genomics of Neurodegenerative Diseases and Aging, Human Genetics, Vrije Universiteit Amsterdam, Amsterdam UMC, Amsterdam, Netherlands; ^8^ Barcelonaβeta Brain Research Center (BBRC), Barcelona, Spain; ^9^ Radboud University Medical Center, Nijmegen, Netherlands; ^10^ Centre for Medical Image Computing, University College London, London, United Kingdom; ^11^ Department of Health Science (DISSAL), University of Genoa, Genoa, Italy; ^12^ IRCCS Ospedale Policlinico San Martino, Genova, Italy; ^13^ Université de Toulouse, Toulouse, France; ^14^ Fundación CITA‐Alzhéimer Fundazioa, Donostia‐San Sebastian, Spain; ^15^ Scottish Brain Sciences, Edinburgh, Scotland, United Kingdom; ^16^ CIBER Bioingeniería, Biomateriales y Nanomedicina (CIBER‐BBN), Madrid, Spain; ^17^ University of Naples Federico II, Naples, Italy; ^18^ Amsterdam UMC, Amsterdam, Netherlands; ^19^ University College London, London, United Kingdom

## Abstract

**Background:**

The Alzheimer's disease (AD) field has shifted toward a biological definition based on the deposition of amyloid‐β1‐42 (Aβ1‐42) and phosphorylated‐Tau181 (*p*‐Tau181), yet only a subset of Aβ1‐42‐positive individuals progresses to AD. The variability in the brain's susceptibility to AD pathology may arise from the effect of polygenic pathways and their interaction with Aβ1‐42 and *p*‐Tau181 deposition. Here, we explored the effect of pathway‐specific polygenic risk scores (PRSs), AD pathology, and their interaction on WM fiber density (FD) and fiber cross‐section (FC) using a fixel‐based approach.

**Method:**

We analyzed 803 non‐demented participants from the European Prevention of Alzheimer's Dementia (EPAD) cohort (age 64.7±7.3 years, 57.0% females, 34.2% APOE4 carriers, Table 1) who underwent genome sequencing, CSF Aβ1‐42 and *p*‐Tau181 measurements, and structural and diffusion MRI. Bellenguez PRSs were constructed using 85 genetic variants associated with AD. After excluding those mapping to *APOE* loci, genetic variants were grouped based on biological function into 6 pathway‐specific PRSs (i.e., amyloid, clearance, immune activation, inflammation, migration and signal transduction pathways). FD and FC were computed from diffusion MRI and averaged within WM bundles. Linear models were used to assess the effect of pathway‐specific PRSs, Aβ1‐42 and *p*‐Tau181, and their interactions on WM, at the bundle and fixel levels. Models were corrected for age, sex, total intracranial volume, and *APOE*‐ε4 carriership. *p*‐values were adjusted for multiple comparisons using the false discovery rate method.

**Result:**

P‐Tau181 showed both increasing and decreasing effects on FD, indicating a multiphasic relationship. The migration pathway was linked to greater FD and FC primarily in the left hemisphere, aligning with hemispheric dominance (Figure 1). Pathology‐dependent effects of pathway‐PRSs were observed. Aβ1‐42 moderated the effect of the clearance pathway on FD, with a positive slope in A+ compared to A‐ individuals (Figure 2A). *p*‐Tau181 moderated the effect of the immune activation pathway on FD, with a negative slope in T+ compared to T‐ individuals (Figure 2B).

**Conclusion:**

Polygenic risk for AD influences WM integrity both directly and via AD pathology‐dependent effects. Genetic susceptibility to preclinical AD pathology activates distinct biological processes that differentially affect WM integrity.